# IGF Signaling in Intervertebral Disc Health and Disease

**DOI:** 10.3389/fcell.2021.817099

**Published:** 2022-02-01

**Authors:** Hui Lin, Shuo Tian, Yizhong Peng, Ling Wu, Yan Xiao, Xiangcheng Qing, Zengwu Shao

**Affiliations:** ^1^ Department of Orthopedics, Union Hospital, Tongji Medical College, Huazhong University of Science and Technology, Wuhan, China; ^2^ Department of Radiology, Union Hospital, Tongji Medical College, Huazhong University of Science and Technology, Wuhan, China

**Keywords:** insulin-like growth factor, intervertebral disc, nucleus pulposus, degeneration, low back pain

## Abstract

Low back pain (LBP) is a common musculoskeletal symptom, which brings a lot of pain and economic loss to patients. One of the most common causes of LBP is intervertebral disc degeneration (IVDD). However, pathogenesis is still debated, and therapeutic options are limited. Insulin-like growth factor (IGF) signaling pathways play an important role in regulating different cell processes, including proliferation, differentiation, migration, or cell death, which are critical to the homeostasis of tissues and organs. The IGF signaling is crucial in the occurrence and progression of IVDD. The activation of IGF signaling retards IVDD by increasing cell proliferation, promoting extracellular matrix (ECM) synthesis, inhibiting ECM decomposition, and preventing apoptosis and senescence of disc cells. However, abnormal activation of IGF signaling may promote the process of IVDD. IGF signaling is currently considered to have a promising treatment prospect for IVDD. An in-depth understanding of the role of IGF signaling in IVDD may help find a novel approach for IVDD treatment.

## Introduction

Intervertebral disc degeneration (IVDD) is a paramount contributor to low back pain and is a leading cause of disability that reduces the quality of life and causes economic loss (Feng et al., 2017; Choi et al., 2019). The structural failure of degenerative discs is marked by disc height collapse, annulus fibrosus (AF) fissures, loss of proteoglycan (PGs) and water in the nucleus pulposus (NP), and cartilage endplate (CEP) calcification (Lappalainen et al., 2014; Silagi et al., 2018; Kim et al., 2021). The prevalence of IVDD is continuously increasing because of the rise in population age (DePalma et al., 2011; Choi et al., 2019). However, the current therapy approach of IVDD treatment is to relieve pain using therapies such as physical therapy, drugs, or interbody fusion surgery (Vadala et al., 2015; Chen et al., 2017). There is still no curative therapy today.

The insulin-like growth factor (IGF) signaling pathways play an important role in regulating various cell activities, including proliferation, differentiation, migration, or cell death, which are critical to the homeostasis of tissues and organs (Duan and Xu, 2005; Rafacho et al., 2009; Lunn et al., 2015). Several investigations have indicated that IGF signaling is critical in the progression of IVDD. Studies have reported that the expressions of IGF are significantly abnormal in degenerative IVD tissues and cells, and it is involved in multiple pathological processes of disc degeneration by participating in cell proliferation, programmed death, degeneration and synthesis of ECM (Tao et al., 2015; Chen et al., 2020; Zhao et al., 2020). IGF signaling is considered to be a promising approach for treatment strategies of IVDD. Here, we review the literature describing how IGF signaling is involved in IVDD pathophysiology and describe recent progression regarding its administration as a promising biological therapeutic approach for disc degeneration.

## IGF Signaling

The IGF axis is composed of ligands such as insulin, insulin-like growth factors 1 and 2 (IGF1, IGF2), receptors (IGF-1R, IGF-2R), IGF binding proteins (IGFBPs) one to seven, and IGFBP protease (Martel-Pelletier et al., 1998; Wang et al., 2014; Hua et al., 2020; Ianza et al., 2021). IGF ligands bind its receptors and binding proteins with high affinity (Huang et al., 2011a; Okamoto et al., 2013). The function of IGFBPs is to bind to IGF, extend the circulating half-life, and prevent the activation of IGF receptors (Patel and Majumdar, 2009; Kobayashi-Hattori et al., 2011). IGFBP protease cleaves IGFBP into fragments with a lower affinity for IGF ligands, thereby improving the bioavailability of free IGF (Peters et al., 2003; Ianza et al., 2021). Three main proteases can cleave IGFBP, including serine proteases, cathepsins, and matrix metalloproteinases (MMPs) (Grimberg and Cohen, 2000; Garcia-Touchard et al., 2005; Chao et al., 2021). The IGF in the intervertebral disc may come from two sources: 1. Circulating IGF1 secreted by the liver; The lack of IGF1 content in plasma can reduce the formation of the IGF1R binding complex in the intervertebral discs by 10–20% (Elmasry et al., 2016). 2. Autocrine/paracrine IGF in the intervertebral disc tissue (Osada et al., 1996; Elmasry et al., 2016). Osada revealed that IGF1 mRNA was expressed in bovine nucleus pulposus cells and annulus fibrosus cells by *in situ* hybridization histochemistry detection. The staining of IGF1R was also positive in the disc cells by the avidin-biotin peroxidase complex method (Osada et al., 1996). Theoretically, with the progression of cartilage degeneration, the cartilage endplate will be calcified, resulting in the deterioration of the nutrition supply to the discs. Such pathological changes will impair the reach of circulating IGF to the intervertebral disc tissue (Urban et al., 2004; Wong et al., 2019). Therefore, the autocrine/paracrine mechanism of IGF in adult nucleus pulposus may be an important source of IGF in degenerative nucleus pulposus. The effects of IGF1 and IGF2 via autocrine/paracrine are mostly mediated by the IGF1 receptors (Kashyap et al., 2020). IGF1R is a heterotetrametric receptor (α2β2) composed of two α and two β subunits which require a series of post-translational modifications, such as glycosylation, disulfide linkage, and proteolytic cleavage, to reach their mature form (Gan et al., 2014; Singh et al., 2014; Kashyap et al., 2020). When the ligand binds to the corresponding receptor, it is activated by autophosphorylation of tyrosine kinases to stimulate IGF signaling pathways (Janssen, 2020; Jozefiak et al., 2021; Lero and Shaw, 2021). The autophosphorylation of tyrosine kinases recruited and activated insulin-receptor substrate 1 (IRS-1) and phosphatidiyl inositol 3 kinase (PI3K), which resulted in the conversion of phosphatidylinositol-3,4,5-trisphosphate 2 (PIP2) to PIP3. The conversion brings PI3-kinase dependent kinase-2 (PDK2) and AKT Serine/Threonine Kinase (Akt) to the membrane and the phosphorylation of Akt activates a number of substrates to regulate protein synthesis, cell proliferation, apoptosis and other cellular activities (Bikle et al., 2015; Yoshida and Delafontaine, 2020; Moonesi et al., 2021). IGF1 can also activate the Grb2 adaptor protein and inducts the activation of Ras G protein to initiate the mitogen-activated kinase (MAPK) pathway, which involves Ras, Raf, MEK and ERK signaling molecules (Clemmons, 2006; Kasprzak, 2021).

**FIGURE 1:**
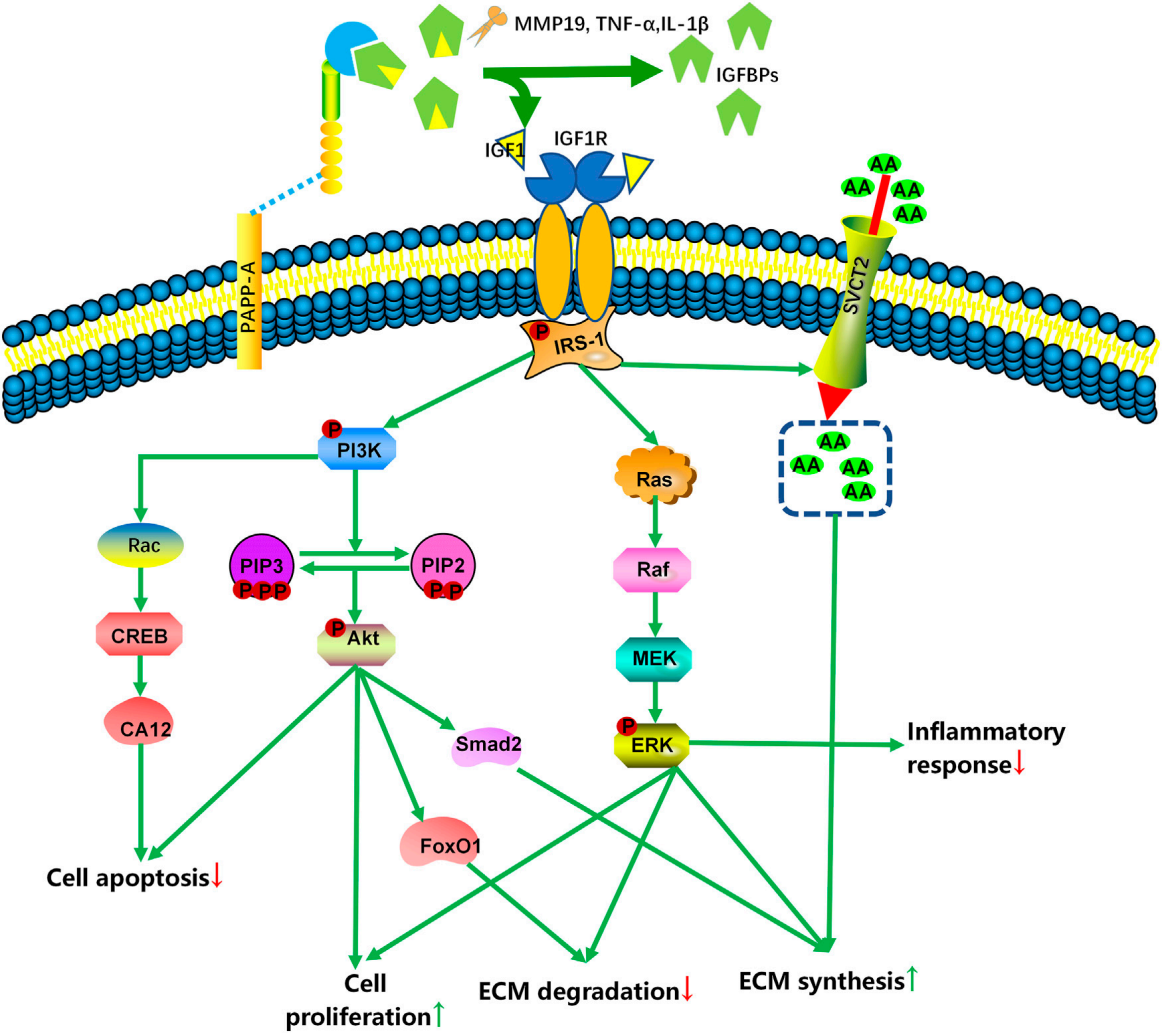
Simplified scheme of IGF signaling in the intervertebral disc. With the participation of MMPs, TNFα, or IL-1β, PAPP-A cleaves IGFBP in the extracellular matrix, increasing the amount of bioavailable IGF1 and IGF2. To exert their effects, IGF bind to specific cell surface receptors (IGF1R and IGF2R). IGF1R activation activates various molecular signaling pathways, including the PI3K-Akt and RAS-extracellular signal-regulated kinase (ERK) pathways, to regulate IVD cell death and ECM content. MMP: matrix metalloproteinases; PAPP-A: pregnancy-associated plasma protein-A; IRS-1: insulin-receptor substrate 1; AA: ascorbic acid; SVCT2: sodium-dependent vitamin C transporter 2; PI3K: phosphatidiyl inositol 3 kinase; PIP: phosphatidylinositol-3,4,5-trisphosphate; FoxO1: forkhead box O1; CREB: cAMP-responsive element-binding protein; CA12: carbonic anhydrase 12; MEK: mitogen-activated protein kinase/extracellular signal-regulated kinase; ERK: extracellular signal-regulated kinase.

IGF1 and its binding protein and receptor system play an important role in the development and degeneration of the intervertebral discs. IGF1 has been shown to affect early dorsal (spine) development, which can promote chondrocyte differentiation and embryonic bone development (Wang et al., 2006). In response to proinflammatory cytokine exposure, Pregnancy-associated plasma protein-A (PAPP-A), a metalloproteinase, cleaves IGFBP in the ECM, making IGF1 available to nearby cells (Gruber et al., 2013; Kritschil et al., 2020). IGF1 is associated with the cell surface receptor tyrosine kinase IGF1R to implement its functions. The binding of the ligand and receptor facilitates the recruitment and phosphorylation of the docking proteins insulin receptor substrates (IRS-1/2), eventually, a series of signaling pathways are triggered (Osher and Macaulay, 2019). Chothe reported that IGF1 upregulated the expressions of the sodium-dependent vitamin C transporter 2 (SVCT2), which facilitate cellular uptake of ascorbic acid from the ECM to maintain the collagen synthesis (Chothe et al., 2013). Zhao et al. unveiled that the activation of the IGF1/PI3K/CREB/CA12 signaling pathway attenuated IVDD by maintaining anabolism and preventing disc cell apoptosis (Zhao et al., 2020). Furthermore, several studies have demonstrated that IGF signaling activation slows IVDD by increasing ECM synthesis, cell proliferation, and inhibiting inflammatory responses, ECM degradation, and cell apoptosis, which are primarily regulated by the PI3K/Akt and MEK/ERK pathways (Pratsinis and Kletsas, 2007; Mavrogonatou and Kletsas, 2010; Pratsinis et al., 2012; Wei et al., 2013; Liu et al., 2015; Tao et al., 2015; Xu et al., 2019; Tian et al., 2020). These evidences indicate that the IGF signaling and its components may be potential new therapeutic targets for this disease (Figure 1).

## The Expression of IGF Signaling in the Intervertebral Discs

Several studies focused on the expression of IGF signaling in the intervertebral discs, and the results showed that it was closely related to IVDD. Osada et al. shown that the expression levels of IGF1 and IGF1R mRNA in the nucleus pulposus of fetal bovine intervertebral discs were higher than those of the adult discs (Osada et al., 1996). However, Murakami found that the mRNA levels of IGF1 in the anulus fibrosus tissue from 3 years old rabbits were 2.0 times higher than in 6 months old rabbits, while in the nucleus pulposus tissue of the old rabbits were only 0.6 times than that in the young rabbits (Murakami et al., 2006). Okuda’s semi-quantitative reverse transcription-polymerase chain reaction research also revealed no evidence of an age-related decrease in IGF1 expression. In terms of IGF1R, there was a significant decrease in 120-week-old cells. IGFBP-1 is expressed more strongly in 40-weeks cells than that in 8-weeks cells (Okuda et al., 2001). There is also still no consensus in the literature about the changes of IGF signaling pathways expression in human IVD tissues. Liu et al. reported that the activation of IGF1R was significantly decreased in degenerated discs (Liu et al., 2015). Chen et al. used ELISA to analyze the levels of IGF1 and IGF1R expression in intervertebral discs with different degrees of degeneration classified according to magnetic resonance imaging and found that IGF1 and IGF1R content was decreased in higher degenerated NP tissues (Chen et al., 2020). However, Le Maitre et al., on the other hand, found that the expression of IGF1R showed no statistically significant differences between non-degenerate and degenerate biopsies (Le Maitre et al., 2005). Tsarouhas et al. also confirmed that the transcriptional levels of the IGF did not differ between the control and herniated groups (Tsarouhas et al., 2017). Interestingly, Specchia et al. discovered that the expression of IGF1 was stronger in herniated discs than in controls (Specchia et al., 2002). Research on the changes in the expression of IGF signaling pathways during the IVDD process is still in its infancy (Table 1). The variation in its expression may be associated with species and different stimulated conditions. One further point to note is that additional measures are needed to detect more IGF signal-related molecules.

**TABLE 1:** The changes in the expression of IGF signaling in the IVDs of different species (NS, not significant).

Species	Classifies	IVD area	Measure methods	Components	Expression changes	Trend	References
Bovine	7 months, 4 years	AF, NP	hybridization histochemistry	IGF1, IGF1R	1. The expression of IGF1 mRNA decreases with ages	↓	Osada et al. (1996)
					2. The IGF1R staining positive cells decreased with ages		
Rabbits	6 months, 3 years	AF,NP	RT-PCR	IGF1	1. The mRNA levels of IGF1 in the anulus fibrosus tissue from the old rabbits were 2.0 times higher than in the young rabbits	↑(AF)	Murakami et al. (2006)
					2. The mRNA levels of IGF1 in the nucleus pulposus tissue of the old rabbits were only 0.6 times that in the young rabbits	↓(NP)	
Rats	6 months, 3 years	NP	Immunohistochemistry	IGF1	1. The IGF1 expression of the test group was higher than in the control group	↑	Xu et al. (2019)
Rats	8, 40, 120 weeks	NP	RT-PCR	IGF1, IGF1R, IGFBP-1, IRS-1	1. No age-related decline in the expression of IGF1 was detected	NS(IGF1)	Okuda et al. (2001)
			Western blot		2. In terms of IGF1R, there was no obvious difference between 8-weeks and 40-weeks cells, whereas an apparent decrease was detected in 120-weeks cells	↓(IGF1R)	
			Immunoprecipitation		3. The expression of IGFBP-1 was not detected in 8-weeks cells, whereas apparent expression was evident in 40-weeks cells, and expression increased with age	↑(IGFBP-1)	
					4. There were no obvious differences in the expression of total IRS-1 among age groups	NS(IRS-1)	
Human	Normal disc, Herniated disc	AF, NP	Immunohistochemistry	IGF1	1. IGF-1 was present in chondrocytes of both normal and pathological tissue, with a stronger labelling in the latter	↑	Specchia et al. (2002)
Human	Normal disc, Herniated disc	AF, NP	Immunohistochemistry	IGF1R	1. There were no significant differences of IGF1R in non- degenerate and degenerate biopsies	NS	Le Maitre et al. (2005)
					2. The expression of IGF1R was observed in the ingrowing blood vessels that characterize part of the disease aetiology		
Human	Normal disc, Herniated disc	Intervertebral disc specimens	Western blot	Phosphorylation of IGF1R	1. The IGF1R was significant deactivated in degenerated discs	↓	Liu et al. (2015)
Human	Normal disc, Herniated disc	iAF, oAF, NP	Immunohistochemistry	PAPP-A	1. The percentage of cells positive for PAPP-A localization did not differ in the human outer AF	NS (oAF)	Gruber et al. (2008)
					2. The percentage of cells positive for PAPP-A localization in more degenerate discs was significantly greater than the percentage in healthier discs in the inner AF	↑(iAF)	
					3. The percentage of cells positive for PAPP-A localization did not differ in the human NP	NS (NP)	
Human	Normal disc, Herniated disc	NP	RT-qPCR	IGF1, IGF2	1. The transcript levels of the IGFs examined were not significant between the control and herniation groups	NS	Tsarouhas et al. (2017)
Human	Denegerated discs (grade 3-5)	NP	ELISA	IGF, IGF1R	1. IGF1 and IGF1R decreased in degenerated human NP tissues	↓	Chen et al. (2020)

## IGF Signaling Inhibits ECM Degradation and Enhances ECM Synthesis

IVD is a complicated joint structure made up of three major components. The IVD’s center, NP, is gelatinous and resilient (Chung et al., 2003; Chou et al., 2016). The AF circumferentially encapsulates the NP while the CEPs are located above and below the NP and AF (Hamilton et al., 2006; Merceron et al., 2014; Kim et al., 2021). The ECM of the NP is composed of collagen, especially type II, non-collagenous proteins, elastin, and proteoglycans (Loreto et al., 2011; Ghannam et al., 2017; Oichi et al., 2020). NP cells are responsible for maintaining the homeostasis and balance of ECM, thereby maintaining the integrity of disc structure and function (Wang et al., 2015b; Zhang et al., 2019). Aggrecan is the most abundant extracellular matrix in nucleus pulposus, which can account for 50% of the dry weight of nucleus pulposus (Le Maitre et al., 2007; Chen et al., 2013; Wang et al., 2015a). Aggrecan can absorb nutrients and water from the periphery through the creation of an osmotic gradient, thus maintaining the hydrophilic nature of NP (Bergknut et al., 2013; Wang et al., 2015a). The turnover of aggrecan in the intervertebral disc is an early sign of IVDD (Sivan et al., 2014). Type II collagen forms a fibrous framework to trap proteoglycans. It has tensile strength and is crucial in the biomechanical function of IVD (Li et al., 2013). In healthy intervertebral discs, due to the complex regulation of growth factors and catabolic factors, the rate of synthesis and decomposition of the extracellular matrix is balanced (Wang et al., 2020). Increased proteolytic degradation of aggregated polysaccharides and a rise in non-aggregated proteoglycans are early degenerative modifications in intervertebral discs (Kim et al., 2021). IVDD occurs when ECM catabolism exceeds anabolism (Guilak et al., 2018). Matrix metalloproteinases (MMPs) and disintegrins with thrombospondin motifs and metalloproteinases (ADAMTs) are important enzymes that cause the loss of the extracellular matrix (Zhang et al., 2021). A variety of ADAMTS and MMPs are up-regulated in degenerative IVD, and they are closely related to the destruction of the ECM and the progression of IVDD (Pockert et al., 2009; Tsarouhas et al., 2011; Huang et al., 2019).

**FIGURE 2:**
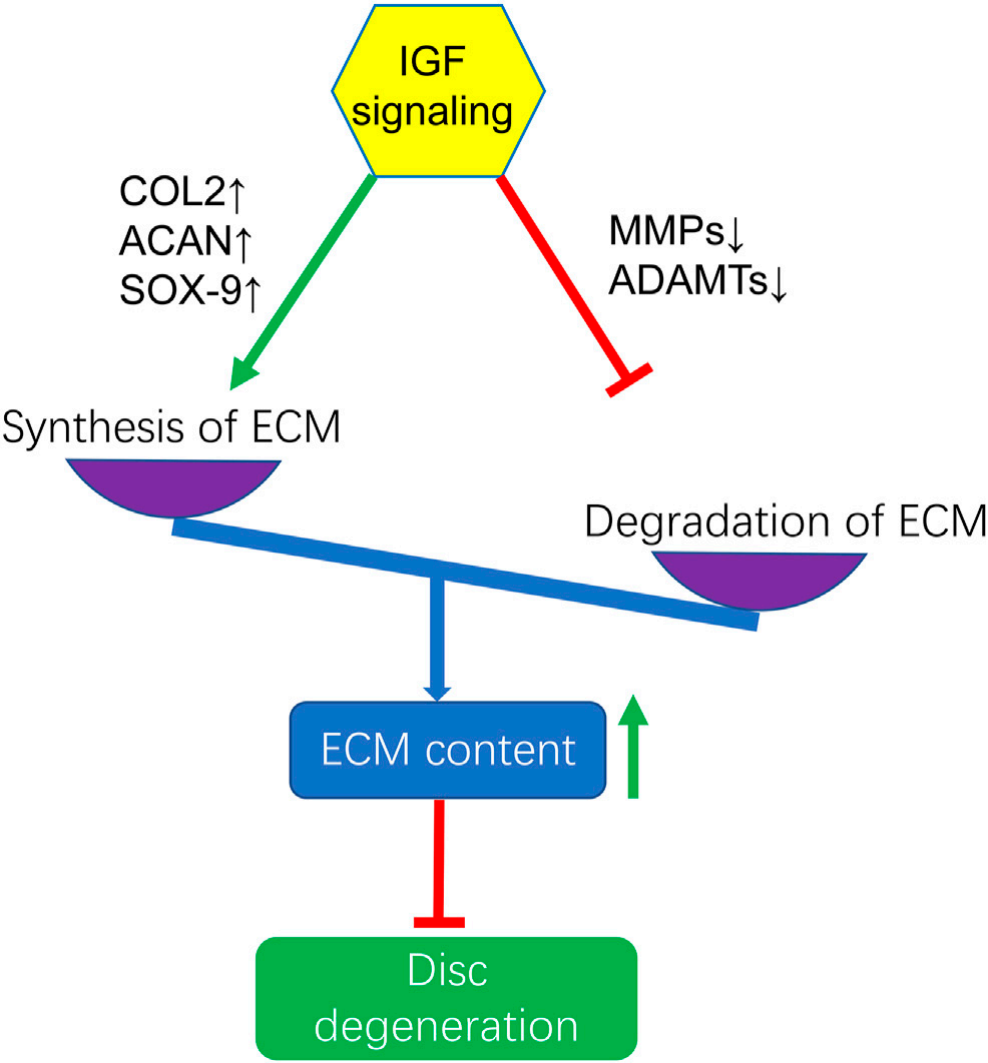
Activation of IGF signaling enhances ECM content by increasing the synthesis of ECM through upregulating the gene expression of COL2, ACAN, SOX-9 and inhibitng the degradation of ECM by downregulating the gene expression of MMPs and ADAMTs to exert protection against IVDD. COL2, Collagen-II; ACAN, Aggrecan.

Several studies have shown that IGF1 can promote the synthesis of ECM (Osada et al., 1996; Gruber et al., 2004; Moon et al., 2008; Kim et al., 2010; Hayes and Ralphs, 2011; Huang et al., 2011b; Illien-Junger et al., 2012; Chothe et al., 2013; Liu et al., 2014; Tao et al., 2015; Chen et al., 2020; Zhao et al., 2020). It also prevents ECM degradation by inhibiting MMPs, which increases the amount of matrix in the intervertebral disc and delays the progression of IVDD. MMP13 is an important enzyme in the degradation of ECM components such as collagen and proteoglycans (Zhang et al., 2009). In animals with IGFIR gene knockout, type II collagen and aggrecan gradually decreased, but the expression of MMP13 mRNA increased in a time-dependent manner (Li et al., 2013). IGF1 treatment significanlty reduced MMP3 expression levels and increased ECM levels in mice degenerated discs conducted by leptin receptor knockout (Li et al., 2020). In addition, *in vitro* studies had found that IGF1 increased the synthesis of proteoglycans and inhibited the production of MMP2 (Pattison et al., 2001). IGF1 can not only inhibit catabolism but also promote anabolism (Osada et al., 1996; Okuda et al., 2001; Pattison et al., 2001; Zhang et al., 2013; Kritschil et al., 2020; Li et al., 2020). IGF1 induces nucleus pulposus mesenchymal stem cells to synthesize ECM by up-regulating the expression of chondrogenic genes COL2, ACAN and SOX-9 *via* the ERK/MAPK signaling pathway (Tao et al., 2015). IGF1 also regulates ECM anabolism by stimulating the production of proteoglycans in ECM (Travascio et al., 2014). Okuda et al. found that the synthesis of proteoglycans in rat nucleus pulposus cells at 8 weeks increased by 4 times compared with the control group. However, in 120-weeks cells supplemented with long R3 IGF1, no significant up-regulation of proteoglycan synthesis was detected. At the same time, the rise in IGFBP-1 in the early stages of aging contributes to the age-related decline in IGF1-dependent proteoglycan production (Okuda et al., 2001). Thus, the therapeutic effects of IGF1 in the increase of ECM are associated with the time of intervention. Simultaneously, we should attach importance to the role of IGF1R and IGFBPs in the regulation of IVDD. In addition, the specific molecular mechanisms of IGF signaling pathways in promoting the synthesis of ECM remain unclear (Figure 2).

## IGF Signaling Promotes Cell Proliferation and Prevents Cell Death

The underlying mechanism of IVDD is disc cell death and loss. Studies have shown that IGF1 can relieve the inhibition of intervertebral disc cells proliferation by the adverse microenvironment (Zhang et al., 2006; Pratsinis and Kletsas, 2007; Mavrogonatou and Kletsas, 2010; Pratsinis et al., 2012; Tao et al., 2015; Akyuva et al., 2019; Chen et al., 2020; Kritschil et al., 2020). Pratsinis and Kletsas found that IGF1 increases IVD cell proliferation via the ERK and Akt signaling pathways (Pratsinis and Kletsas, 2007). Eleni et al. also reported that IGF1 stimulated DNA synthesis in NP cells under different osmotic conditions via the ERK and Akt signaling pathways (Mavrogonatou and Kletsas, 2010). Similarly, in 10% fetal bovine serum, IGF1 at a concentration of 100 μg/L significantly increased cell proliferation (Zhang et al., 2006). This evidence indicates that IGF plays a positive regulatory role in promoting cell proliferation. However, the hyper-proliferation of disc cells leads to a rise in nutrition consumption and demand, which has been implicated in IVDD (Guo et al., 2019). Therefore, the pro-proliferative effects of IGF are not necessarily to retards the process of IVDD. More research is required to explore the effects of IGF signaling on the degeneration of intervertebral discs in regulating cell proliferation in different periods.

As to programmed cell death, growing evidence indicates that IGF could retard cell apoptosis to delay IVDD (Gruber et al., 2000; Price et al., 2007; Liang et al., 2012; Zhang et al., 2013; Kuo et al., 2014; Li et al., 2020; Zhao et al., 2020). A study showed that the administration of IGF1 significantly reduced IL-1β-induced apoptosis of NP cells (Zhang et al., 2013). Type II diabetes mellitus (T2DM) induced by leptin receptor knockout in mice led to IVDD by promoting disc cell apoptosis. IGF1 treatment partially reversed this situation (Li et al., 2020). Gruber proved that after exposure to 500 ng/ml IGF1, the percentage of intervertebral disc cell apoptosis was significantly reduced (Gruber et al., 2000). NP cells treated with an adenoviral vector expressing hIGF1 showed a decreased rate of apoptosis confirmed by the TUNEL test and FCM conducted by TNFα (Zhang et al., 2014). In addition, IGF1 can also inhibit cellular senescence caused by H_2_O_2_ (Gruber et al., 2008). In summary, these results indicate that IGF1 delays IVDD by inhibiting cell apoptosis. It is worth noting that although much published literature has confirmed that IGF1 can inhibit the apoptosis of disc cells caused by various unfavorable stimulation, there is still a lack of research on its specific signaling pathways. In addition, studies have confirmed that the degenerated intervertebral disc cells are mainly necrotic-like changes (Buckwalter, 1995). However, the effects of IGF on the programmed necrosis of NP cells have not been reported yet.

## Abnormal Activation of IGF Signaling Aggravates IVDD

IGF signaling is involved in various diseases and is considered to have pleiotropic effects (Dixit et al., 2021). It has recently been reported that the over-activation of IGF signaling may contribute to the progress of IVDD. In the case of pathological nutritional insufficiency, exogenous injection of IGF1 is only beneficial to well-nourished areas in IVD, while in undernourished areas will increase cell mortality (Travascio et al., 2014). In addition, IGF1 promotes cell proliferation by increasing the production of PG and promoting cell metabolism, thereby increasing the nutritional requirements of the intervertebral discs (Pratsinis and Kletsas, 2007; Huang et al., 2012). This may further promote intervertebral disc degeneration because insufficient nutritional supply is considered to be the main cause of IVDD (Travascio et al., 2014). The injection of IGF in human IVD may induce unnecessary vascular ingrowth and accelerate the process of IVDD (Li et al., 2013). With an advancing grade of disc degeneration, enhancement of angiogenesis is usually accompanied by nerve ingrowth, producing painful discs (Le Maitre et al., 2005). The angiogenic potential of IGF may avail the ingrowth of blood vessels and bring painful discs (Le Maitre et al., 2005). Takayama et al. revealed that IGF1 knockdown resulted in a relief of mechanical allodynia in the dorsal root ganglion cells of a rat model of disc herniation (Takayama et al., 2011). Koerner collected intervertebral discs from patients who had undergone lumbar interbody fusion surgery for back pain and determined the expression levels of IGF1. The results indicated that IGF1 significantly increased in the posterior AF versus the anterior AF. Nonetheless, there were no differences in the anterior AF compared with posterior AF in the scoliosis group. The overexpression of growth factors may predispose the posterior AF to disc degeneration, compromise the structural properties of the discs and is associated with facet arthritis and discogenic pain (Koerner et al., 2014). In addition, the ultimate therapeutic effect of IGF1 varies as the concentration change. In 10% fetal bovine serum, IGF1 at a concentration of 100 μg/L significantly increased cell proliferation and showed a dose-dependent effect. However, the viability of NP cells declined as the concentration increased (Zhang et al., 2006). The activation of IGF1 leads to increased expression of IL-1 and IL-2 via the PI3K/Akt signaling pathway in herniated lumbar discs (Xu et al., 2019). These results indicate that the abnormal activation of IGF signaling pathways may accelerate the process of IVDD (Table 2).

**TABLE 2:** Abnormal activation of IGF signaling aggravates IVDD.

Author	Conclusion	References
Travascio et al	Exogenous injection of IGF1 is only beneficial to well-nourished areas in IVD, while in undernourished areas will increase cell mortality	Travascio et al. (2014)
Li et al	The injection of IGF in human IVD may induce unnecessary vascular ingrowth and accelerate the process of IVDD.	Li et al. (2013)
Le Maitre et al	The angiogenic potential of IGF may avail the ingrowth of blood vessels and bring painful discs	Le Maitre et al. (2005)
Takayama et al	IGF1 knockdown resulted in a relief of mechanical allodynia in the dorsal root ganglion cells of a rat model of disc herniation	Takayama et al. (2011)
Koerner et al	High expression of IGF1 may be related to the pain experienced in IVDD.	Koerner et al. (2014)
Zhang et al	IGF1 at a concentration of 100 μg/L significantly increased cell proliferation but the viability of NP cells declined as the concentration increased	Zhang et al. (2006)
Xu et al	The activation of IGF1 lead to increased expression of IL-1 and IL-2 via the PI3K/Akt signaling pathway in herniated lumbar discs	Xu et al. (2019)

## Therapeutic Effects of IGF Signaling in IVDD

Both *in vitro* and *in vivo* studies have exhibited that IGF signaling may be a potential therapeutic target for the treatment of IVDD (Gruber et al., 2000; Gruber et al., 2001; Gruber et al., 2004; Moon et al., 2008; Mavrogonatou and Kletsas, 2010; Zhang et al., 2013; Akyuva et al., 2019; Zhao et al., 2020). The application of IGF for treating IVDD is mainly achieved through two different approaches: IGF injection into degenerative intervertebral discs and mesenchymal stem cell (MSC) transplantation (Feng et al., 2015). The results of these studies demonstrated promising effects (Feng et al., 2015). The administration of IGF may be helpful to retard disc degeneration by increasing cell proliferation, stimulating matrix synthesis, and inhibiting programmed cell death. For example, IGF injection into the intervertebral discs can effectively inhibit cell apoptosis and matrix degradation in degenerated discs in leptin receptor-deficient knockout mice model (Li et al., 2020). The addition of IGF1 to the culture of intervertebral disc cells has been shown to prevent the senescence of annulus fibrosus (AF) cells and promote the synthesis of proteoglycan in NP cells (Gruber et al., 2008). IGF1 treatment is considered to be a strategy to inhibit IVD degeneration in under T2DM conditions (Mahmoud et al., 2020). In recent years, tissue engineering and gene therapy technologies can provide sustained release of growth factors (Zhang et al., 2006). For example, studies have shown that injection of Ad/CMV-hIGF1 vector as gene therapy has more beneficial effects on rabbit IVDD cells than an injection of hIGF1 alone (Zhang et al., 2014). However, human IVDD is a multifactorial pathological process. The results obtained from *in vitro* and animals experements do not necessarily apply to humanity. Further research is needed to explore the exact role of IGF signaling in the treatment of IVDD using human disc cells.

In recent years, the application of stem cells combined with IGF has provided new ideas for IVDD treatment strategies. Studies have confirmed that IGF1 helps MSC to differentiate into NP-like phenotypes (Ehlicke et al., 2010; Illien-Junger et al., 2012; Chon et al., 2013; Liu et al., 2014; Tao et al., 2015; Kennon et al., 2018; Tian et al., 2020). These NP-like cells could be transplanted into degenerative discs to increase the number of functional cells in IVD or enhance the function of endogenous intervertebral disc cells (Shim et al., 2016). Under conditions of hypoxia and nutrient deficiency, adding IGF1 to hNP-MSCs can improve cell proliferation and prevent the decline of matrix gene expression, and inhibit cell apoptosis (Tian et al., 2020). The homing of bone marrow mesenchymal stem cells transduced with IGF1 can accelerate the synthesis of proteoglycan in degenerated intervertebral discs (Illien-Junger et al., 2012).

Although many studies have confirmed the promising results of the application of IGF in the treatment of IVDD, there are still challenges that limit its clinical application. The effects of IGF on cell proliferation, death, and matrix synthesis in intervertebral discs are complicated. The specific role of IGF signaling on intervertebral disc degeneration may be related to the dose and the time of application. Inappropriate use of IGF signaling may aggravate IVDD and contribute to the exacerbations of LBP. In the case of pathological nutritional insufficiency, exogenous injection of IGF1 is only beneficial to well-nourished areas in IVD, while in undernourished areas will increase cell mortality (Travascio et al., 2014). The overexpression of IGF1 in posterior AF positively correlates with the pain experience among patients with IVDD (Koerner et al., 2014). Thus the IGF1 injection to this area may worsen the pain. In addition, PAPP-A showed a complex regulation of IVDD during aging. Deactivation of PAPP-A retarded disc cellular senescence and matrix catabolism but also inhibited matrix anabolism (Kritschil et al., 2020). A comprehensive understanding of IGF signal transduction and the related regulatory factors will contribute to developing better therapies for the treatment of IVDD. However, most patients may have severe intervertebral disc degeneration at the time of diagnosis, whereas IGF may have a better therapeutic effect in the early stage of degeneration. Although IGF may stimulate the endogenous repair mechanism, it may cause further disc damage due to the operation. The half-life of IGF ranges from 10 s to 2 min. This limits its biological effects *in vivo* and restricts its applicability (Akyuva et al., 2019). There are several small molecules that have been developed to target the IGF signaling for the treatment of different diseases. A 14-residue peptidomimetic of IGF1, bp-1-101, which was able to block the IGF/IGFBP interactions, has been proposed as a therapeutic agent for stroke treatment (Rosenzweig, 2004). Natural metabolites of IGF1, glycine-proline-glutamate, cyclic glycine-proline as well as the structural analogues glycine-2-methyl-proline-glutamate and cyclo-L-glycyl-L-2-allylproline exhibited valid neuroprotection (Guan et al., 2015). Small molecules targeting the IGF signaling provide a novel strategy for the treatment of IVDD. However, there are no reports about the clinical applications of small molecules targeting IGF signaling on IVDD at present. The side effects produced by excessive activation or deactivation of IGF signaling may be main obstacles or peoblems in the clinical management. Research on the dose, frequency, and safety of IGF injection is still lacking. A follow-up issue that needs to be addressed in the future is to determine the ideal timing of the interventions. In a word, the application of IGF for IVD regeneration appears promising. Nevertheless, further basic research is still needed before clinical use (Kennon et al., 2018).

## Conclusion

**FIGURE 3:**
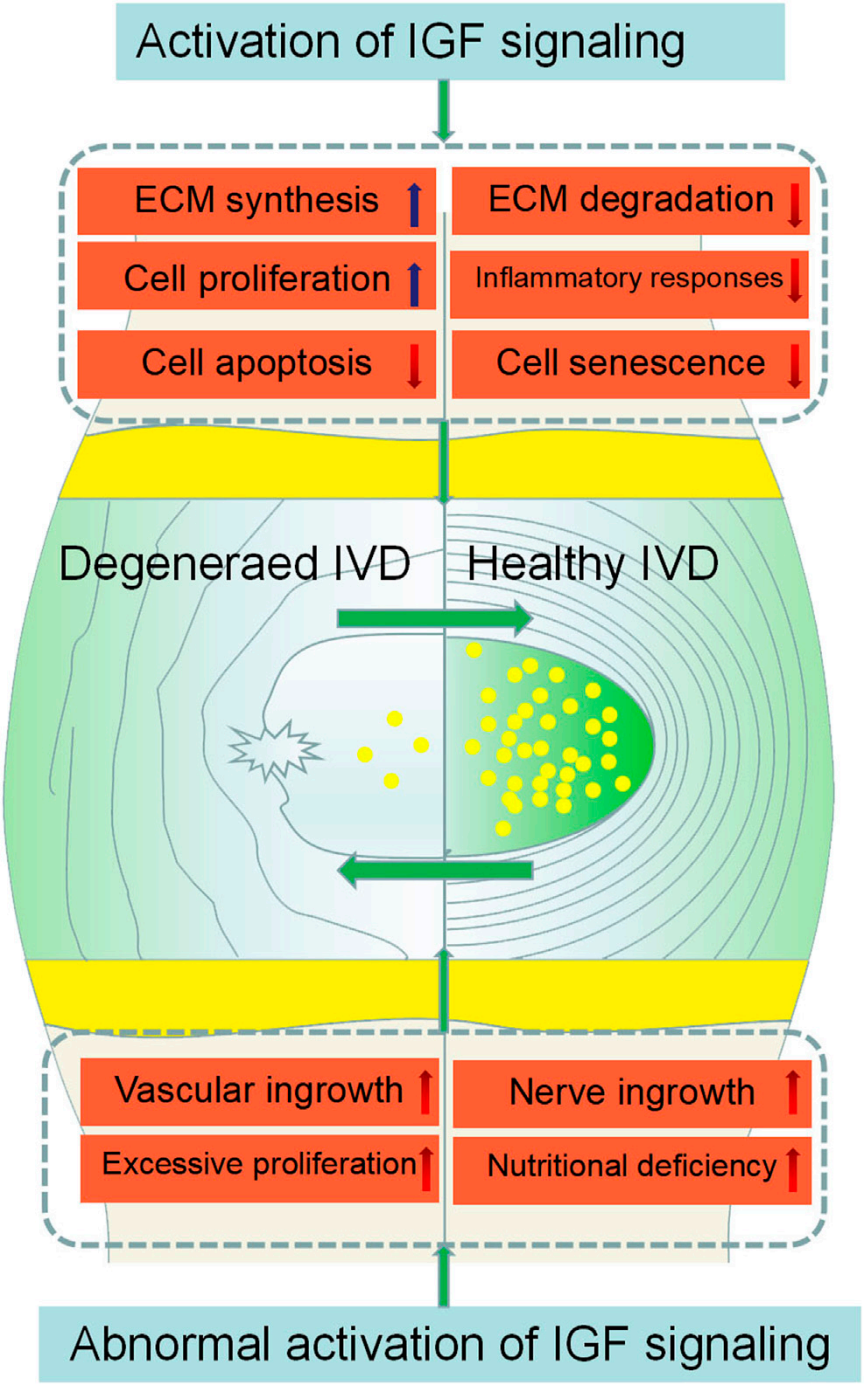
Effects of IGF signaling on IVDD initiation and progression. IGF signaling activation inhibits IVDD by promoting ECM synthesis, cell proliferation, and inhibiting inflammatory responses, ECM degradation, and cell apoptosis. However, the abnormal activation of IGF signaling can accelerate the degeneration of IVD by stimulating excessive cell proliferation, increasing vascular and nerve growth, aggravating nutritional deficiency.

IVDD is the leading contributor to LBP which causes great economic loss and disability globally. Emerging evidence has highlighted the important roles of IGF signaling in the pathogenesis of IVDD. However, the precise mechanisms remain unclear. The activation of IGF signaling can retard IVDD by inhibiting cell apoptosis, cell senescence, matrix degradation, inflammatory and promoting cell proliferation, matrix synthesis. Unfortunately, the abnormal activation of IGF signaling can accelerate IVDD by stimulating excessive cell proliferation, increasing vascular and nerve growth, aggravating nutritional deficiency (Figure 3). Thus there are still a variety of challenges to address when we perform IGF-centered therapy for IVDD. In addition, there is still no ideal way to make drugs reach the intervertebral discs safely and effectively and the studies performed in patients are still lacking. It is urgent to elaborate on the application of IGF signaling in clinical trials to optimize the IGF-based therapy for IVDD treatment.
