# Supplementary material for: IGF Signaling in Intervertebral Disc Health and Disease
Source: Front Cell Dev Biol. 2022 Feb 1;9:817099. doi: 10.3389/fcell.2021.817099 (PMC8843937; doi:10.3389/fcell.2021.817099)
Supplement: Supplementary file 1 [file Table1.DOCX]

| Table 1: The changes in the expression of IGF signaling in the IVDs of different species (NS, not significant) | | | | | | | |
| --- | --- | --- | --- | --- | --- | --- | --- |
| Species | Classifies | IVD area | Measure methods | Components | Expression changes | Trend | Reference |
| Bovine | 7 months, 4 years | AF, NP | hybridization histochemistry | IGF1, IGF1R | 1. The expression of IGF1 mRNA decreases with ages 2. The IGF1R staining positive cells decreased with ages | ↓ | Osada et al.,1996 |
| Rabbits | 6 months, 3 years | AF,NP | RT-PCR | IGF1 | 1. The mRNA levels of IGF1 in the anulus fibrosus tissue from the old rabbits were 2.0 times higher than in the young rabbits 2. The mRNA levels of IGF1 in the nucleus pulposus tissue of the old rabbits were only 0.6 times that in the young rabbits | ↑(AF)  ↓(NP) | Murakami et al.,2006 |
| Rats | 6 months, 3 years | NP | Immunohistochemistry | IGF1 | 1. The IGF1 expression of the test group was higher than in the control group | ↑ | Xu et al.,2019 |
| Rats | 8, 40, 120 weeks | NP | RT-PCR  Western blot  Immunoprecipitation | IGF1, IGF1R, IGFBP-1, IRS-1 | 1. No age-related decline in the expression of IGF1 was detected. 2. In terms of IGF1R, there was no obvious difference between 8-week and 40-week cells, whereas an apparent decrease was detected in 120-week cells. 3. The expression of IGFBP-1 was not detected in 8-week cells, whereas apparent expression was evident in 40-week cells, and expression increased with age 4. There were no obvious differences in the expression of total IRS-1 among age groups | NS(IGF1)  ↓(IGF1R)  ↑(IGFBP-1)  NS(IRS-1) | Okuda et al.,2001 |
| Human | Normal disc, Herniated disc | AF, NP | Immunohistochemistry | IGF1 | 1. IGF-1 was present in chondrocytes of both normal and pathological tissue, with a stronger labelling in the latter. | ↑ | Specchia et al.,2002 |
| Human | Normal disc, Herniated disc | AF, NP | Immunohistochemistry | IGF1R | 1. There were no significant differences of IGF1R in non- degenerate and degenerate biopsies. 2. The expression of IGF1R was observed in the ingrowing blood vessels that characterize part of the disease aetiology. | NS | Le Maitre et al.,2005 |
| Human | Normal disc, Herniated disc | Intervertebral disc specimens | Western blot | Phosphorylation of IGF1R | 1. The IGF1R was significant deactivated in degenerated discs | ↓ | Liu et al.,2015 |
| Human | Normal disc, Herniated disc | iAF, oAF, NP | Immunohistochemistry | PAPP-A | 1. The percentage of cells positive for PAPP-A localization did not differ in the human outer AF 2. The percentage of cells positive for PAPP-A localization in more degenerate discs was significantly greater than the percentage in healthier discs in the inner AF 3. The percentage of cells positive for PAPP-A localization did not differ in the human NP | NS (oAF)  ↑(iAF)  NS (NP) | Gruber et al.,2008 |
| Human | Normal disc, Herniated disc | NP | RT-qPCR | IGF1, IGF2 | 1. The transcript levels of the IGFs examined were not significant between the control and herniation groups | NS | Tsarouhas et al.,2017 |
| Human | Denegerated discs (grade 3-5) | NP | ELISA | IGF, IGF1R | 1. IGF1 and IGF1R decreased in degenerated human NP tissues | ↓ | Chen et al.,2020 |
