# Supplementary material for: IGF Signaling in Intervertebral Disc Health and Disease
Source: Front Cell Dev Biol. 2022 Feb 1;9:817099. doi: 10.3389/fcell.2021.817099 (PMC8843937; doi:10.3389/fcell.2021.817099)
Supplement: Supplementary file 2 [file Table2.DOCX]

| Table 2: Abnormal activation of IGF signaling aggravates IVDD | | |
| --- | --- | --- |
| Author | Conclusion | Reference |
| Travascio et al. | Exogenous injection of IGF1 is only beneficial to well-nourished areas in IVD, while in undernourished areas will increase cell mortality. | Travascio et al., 2014 |
| Li et al. | The injection of IGF in human IVD may induce unnecessary vascular ingrowth and accelerate the process of IVDD. | Li et al., 2013 |
| Le Maitre et al. | The angiogenic potential of IGF may avail the ingrowth of blood vessels and bring painful discs. | Le Maitre et al., 2005 |
| Takayama et al. | IGF1 knockdown resulted in a relief of mechanical allodynia in the dorsal root ganglion cells of a rat model of disc herniation. | Takayama et al., 2011 |
| Koerner et al. | High expression of IGF1 may be related to the pain experienced in IVDD. | Koerner et al., 2014 |
| Zhang et al. | IGF1 at a concentration of 100 μg/L significantly increased cell proliferation but the viability of NP cells declined as the concentration increased. | Zhang et al., 2006 |
| Xu et al. | The activation of IGF1 lead to increased expression of IL-1 and IL-2 via the PI3K/Akt signaling pathway in herniated lumbar discs. | Xu et al., 2019 |
